# Investigation of the Genotoxic, Antigenotoxic and Antioxidant Profile of Different Extracts from *Equisetum arvense* L.

**DOI:** 10.3390/antiox11071393

**Published:** 2022-07-18

**Authors:** Margarita Dormousoglou, Ioanna Efthimiou, Maria Antonopoulou, Damian L. Fetzer, Fabiane Hamerski, Marcos L. Corazza, Maria Papadaki, Samir Santzouk, Stefanos Dailianis, Dimitris Vlastos

**Affiliations:** 1 Department of Environmental Engineering, University of Patras, Seferi 2, GR-30100 Agrinio, Greece; m.dormousoglou@upatras.gr (M.D.); iefthimiou@upatras.gr (I.E.); mantonop@upatras.gr (M.A.); marpapadaki@upatras.gr (M.P.); 2 Hellenic Centre for Marine Research (HCMR), Institute of Marine Biology, Biotechnology and Aquaculture, Anavyssos, GR-19013 Athens, Greece; 3 Department of Chemical Engineering, Federal University of Paraná, Curitiba 81531-990, Brazil; fetzer@ufpr.br (D.L.F.); fabianehamerski@ufpr.br (F.H.); corazza@ufpr.br (M.L.C.); 4 Santzouk Samir and Co. General Partnership, PANAX, Chrissostomou Smirnis 14, GR-30100 Agios Konstantinos, Greece; smsamir@otenet.gr; 5 Department of Biology, University of Patras, GR-26500 Patras, Greece; sdailianis@upatras.gr

**Keywords:** *Equisetum arvense* L., CBMN assay, cyto-genotoxicity, antigenotoxicity, Soxhlet extraction, antioxidant activity, human lymphocytes

## Abstract

The present study investigated the cyto-genotoxic and antigenotoxic effects of four different extracts of *Equisetum arvense* L. (common name: field horsetail) on human lymphocytes. Specifically, Soxhlet’s prepared extracts from *E. arvense* L., using different solvents (S1: methanol (MeOH)-, S2: ethanol (EtOH)-, S3: water-, and S4: ethanol/water (EtOH-W)-) were analyzed for (a) their total phenolic and flavonoid content (TPC and TFC, respectively), (b) their antioxidant activity (AA), via the DPPH, FRAP and ABTS assays, and (c) their cyto-genotoxic and/or protective efficiency against the mutagenic agent mitomycin C, via the Cytokinesis Block MicroNucleus assay. All extracts showed increased TPC, TFC, and AA values in almost all cases. S1, S3 and S4 demonstrated no cytotoxic potential, whereas S2 was cytotoxic only at the highest concentrations. Genotoxicity was not observed in the tested extracts. The highest antigenotoxic activity was observed for EtOH-W (S4) extract, which was found to be rich in flavonoids, flavonoid-*O*-glycosides, phytosterols, phenolic and fatty acids as well as in minerals and mainly in K, Ca, Mg, Si and P, as assessed by using various mass spectrometry techniques. Those findings confirm that *E. arvense* L. extracts could be valuable candidates for medicinal applications and pharmaceutical products, thus alleviating the effects of more conventional drugs.

## 1. Introduction

Medicinal plants and their extracts constitute potential candidates for novel drugs and therapeutic purposes due to their beneficial properties [1] and have been widely used as food ingredients as well as for the treatment of various ailments [2]. Among them, *E. arvense* L. (field horsetail), belonging to the *Equisetaceae* family, is of great interest due to its beneficial properties and chemical composition.

*E. arvense* L., characterized by worldwide distribution [3,4,5], contains plenty of biologically active compounds such as flavonoids, phenolic acids, alkaloids, phytosterols, tannins and triterpenoids amongst others [6,7]. These compounds possess antioxidant, anti-inflammatory, antidiabetic, antibacterial, antifungal, anticonvulsant, and anticancer properties according to previous studies [3,4,6]. For instance, it is well known that phenolic compounds and flavonoids are effective antioxidants with free radical scavenging activity [8], while other bioactive compounds, such as pigments, anthocyanins, glucosinolates, and tannins, could have a protective effect against prooxidants that could promote the generation of free radicals and the concomitant induction of DNA damage [9]. To this end, their extraction from different parts of medicinal plants using ethanol, methanol and/or water is of great importance [10].

Considering the latter, the present study aimed to investigate *E. arvense* L. beneficial effects by means of the cyto-genotoxic and antigenotoxic potential of four different extracts of its aerial parts on human lymphocytes. Specifically, Soxhlet’s prepared extracts from *E. arvense* L., using different solvents (S1: methanol (MeOH), S2: ethanol (EtOH), S3: water, and S4: ethanol/water (EtOH-W)) were analyzed for their total phenolic and flavonoid content (TPC and TFC, respectively) and their antioxidant activity (AA), via the DPPH, FRAP and ABTS assays. In particular, the Soxhlet extraction constitutes one of the most widely applied methods for the extraction of bioactive compounds from natural products, using appropriate solvents, boiling temperature, and ambient pressure. It is a simple extraction method that provides high extraction yields without affecting the bioactivity of the extracted molecules [11,12]. Afterwards, the cyto-genotoxic potential of each extract, as well as their protective efficiency against the mutagenic agent mitomycin C (MMC), at a final concentration of 0.5 μg mL^−1^ were assessed in cultured human lymphocytes, primarily treated with different concentrations of each extract (10, 50 and 100 μg mL^−1^) via the Cytokinesis Block MicroNucleus (CBMN) assay. MMC was selected as a mutagenic and/or genotoxic inducer since it has been used for this purpose in various antigenotoxicity studies [13,14,15]. MMC has been found to be genotoxic in all in vitro and in vivo test systems in mammalian cells and animals and was clearly demonstrated as a carcinogenic agent [16]. In addition, the usage of MMC as a positive control is proposed by the Organisation for Economic Cooperation and Development (OECD) protocol during the application of the CBMN assay [17]. Finally, the most beneficial extract (S4, EtOH-W) was fully characterized using various mass spectrometry techniques. 

## 2. Materials and Methods

### 2.1. Chemicals and Reagents

Fetal bovine serum (FBS), Ham’s F-10 medium, and Phytohaemaglutinin (PHA) were commercially procured by Gibco (Fisher Scientific, Loughborough, Leicestershire, UK Ltd). Mitomycin C (MMC) and Cytochalasin-B (Cyt-B) were purchased from Sigma-Aldrich Chemical Co. (St. Louis, MO, USA). All other chemicals and solvents were of the highest grade commercially available. Stocks of the compounds and solutions were stored at 4 °C until use. Treatments were performed and based on the initial stocks of the tested compounds.

### 2.2. Sample Preparation

*E. arvense* L. was collected from Aitoloakarnania region, Western Greece (Coordinates: 38°55′36.9″ N 21°24′33.9″ E) in August 2019, and immediately transferred to the laboratory for further analysis. The plant was identified by Assoc. Professor Maria Panitsa, Department of Biology, Section of Plant Biology, University of Patras. Moreover, the voucher specimen of *E. arvense* L. (39894 UPA-Herbarium, Patras, Greece) was deposited for reference purposes in the Herbarium of the Department of Biology of the University of Patras.

Specifically, the aerial parts of the plant were dried at 40 °C for 24 h in a forced air oven, and the final moisture content was determined according to the method of the Association of Official Analytical Chemists (AOAC International: Gaithersburg, MD, USA) [18], where the measured value was 11.5%. Dried samples were ground in a domestic food processor for 10 s and the average particle size was estimated with the method described by Gomide [19] using Tyler series sieves with openings of 8, 10, 20, 28, 32 and 35 mesh in a vertical vibratory sieve shaker. The average particle size (PS) obtained was 0.57 ± 0.02 mm. The raw material was packed in plastic bags and stored at −20 °C until use.

### 2.3. Soxhlet Extraction

Soxhlet extraction was carried out using 5 g of dried plant and 150 mL of solvent according to the method of AOAC [18]. After the addition of the solvent the system was heated until boiling. The extraction lasted for 6 h. Methanol, ethanol, water, and ethanol/water (ratio 4:1) were used as solvents and the procedure was performed in triplicate. Afterwards, the final extracts were concentrated in a rotary vacuum evaporator (Ika RV 10) and subsequently dried using an air circulation oven (IKA, Nova Ιtica, model 400-2; IKA^®^-Werke GmbH & Co. KG, Germany). The extraction yields for each extract (S1: extract obtained with methanol-, S2: extract obtained with ethanol-, S3: extract obtained with water-, and S4: extract obtained with ethanol/water-) were calculated using the following equation:

$$Yield\left(\%\right)=\frac{massofdriedextract\;\left(g\right)}{massofsample\;\left(g\right)}\times 100$$

### 2.4. Determination of Total Phenolic Content, Total Flavonoid Content and Antioxidant Activity

#### 2.4.1. Preparation of Samples

All samples were prepared and analyzed in triplicate. A total of 0.2 mL of extracts S1–S4 were mixed with 0.8 mL of methanol:H_2_O (80:20 *v*/*v*). Afterwards, the mixture was vigorously shaken for 5 min and centrifuged (3000 rpm, 10 min). The supernatant was used to determine the total phenolic content (TPC), total flavonoid content (TFC) and the antioxidant activity (AA).

#### 2.4.2. Total Phenolic Content (TPC)

The determination of the TPC was performed by the Folin–Ciocalteu method according to the procedure described by Singleton et al. [20]. Specifically, aliquots of S1–S4 extracts combined with methanol:H_2_O to reach 0.5 mL in volume were mixed with 2.5 mL of Folin–Ciocalteu reagent (diluted 1:10 in distilled water) and kept in the dark for 3 min. Afterwards, 2 mL of 7.5% Na_2_CO_3_ was added and the mixtures were incubated in the dark for 2 h. Finally, the absorbance was measured at 760 nm (using a UV-Vis spectrophotometer, Global Analitik, Ankara, Turkey). The quantitative results were calculated using an analytical curve of gallic acid. The gallic acid was used as a standard reference and the results were expressed as milligrams of gallic acid equivalents (GAE) per 1 g of extract (mg GAE/g^−1^) ± standard deviation. All runs were performed in triplicate.

#### 2.4.3. Total Flavonoid Content (TFC)

The determination of the TFC was based on the method proposed by Zhishen et al. [21], with some modifications. In brief, 0.2 mL aliquots of samples, combined with 1.8 mL of distilled water and 0.12 mL of NaNO_2_ (5% *w*/*v*), were added to amber bottles and mixed for 5 min. Thereafter, 0.12 mL of AlCl_3_ (10% *w*/*v*) was added, followed by 0.8 mL of NaOH (1 mol L^−1^) and 0.96 mL of dH_2_O. Absorbance was measured spectrophotometrically after 5 min at 510 nm. A catechin calibration curve was used for the expression of the results as mg of catechin equivalent (CE) per 1 g of sample (mg CE g^−1^).

#### 2.4.4. ABTS Radical Cation Decolorization Assay

The ABTS (2,2-azino-bis-(3-ethylbenzotiazoline-6-sulfonic acid)) method was performed according to the procedure described by Re et al. [22]. Τhe ABTS reagent was dissolved in water to a final concentration of 7 mmol L^−1^. The solution (5 mL) was subsequently mixed with 88 μL potassium persulfate solution (140 mmol L^−1^) and then incubated in the dark, at room temperature, for 16 h, to produce a stock solution of the radical cation (ABTS^•+^). For the preparation of the ABTS^•+^ working solution, the stock solution was diluted with absolute ethanol until reaching an absorbance of 0.700 ± 0.020 at 734 nm. In order to analyze the samples, appropriate aliquots of methanol:H_2_O solutions were prepared as previously described; methanol up to 100 μL was added to amber bottles and mixed with 3.9 mL of the ABTS^•+^ radical cation working solution (A_734nm_ = 0.700 ± 0.020). The absorbance of the resulting solution was measured at 734 nm, 6 min after the initial mixing of the samples with the ABTS solution. Quantification was conducted using a Trolox analytical curve, and the results were expressed as µmol of Trolox equivalents per 1 g of sample (μmol TE g^−1^).

#### 2.4.5. Radical Scavenging Activity by DPPH^•^ Assay

The DPPH^•^ assay was performed based on the method described by Brand-Williams et al. [23]. A 3.9 mL aliquot of a DPPH^•^ methanolic solution (60 mM or 6 × 10^−5^ mol L^−1^) was mixed with 100 μL of diluted samples. After being incubated for 1 h in the dark, the DPPH^•^ absorbance was measured at 515 nm. A Trolox analytical curve was used for quantification and the results were expressed as μmol of Trolox equivalents per 1 g of sample (μmol TE g^−1^).

#### 2.4.6. Ferric Reducing Antioxidant Power (FRAP) Assay

The FRAP assay was conducted according to Benzie and Strain [24]. Briefly, 200 μL of each diluted sample was mixed with 200 μL of water and 3 mL of FRAP reagent. The FRAP reagent consisted of a mixture of 300 mmol L^−1^ sodium acetate buffer solution (pH 3.6), 10 mmol L^−1^ of TPTZ solution in 40 mmol L^−1^ HCl and 20 mmol L^−1^ FeCl_3_ solution in a volume ratio of 10:1:1. Following the addition of the FRAP reagent, the flasks were placed in a water bath at 37 °C for 10 min. The absorbance of the mixture was measured 15 min after the incubation period at 593 nm. For the quantification step, a Trolox analytical curve was constructed, and the results were expressed as μmol of Trolox equivalents per 1 g of sample (μmol TE g^−1^).

### 2.5. CBMN Assay

#### 2.5.1. Ethics Statement

The CBMN assay using human lymphocytes was carried out in accordance with international bioethics criteria, after the permission/approval of the Research Ethics Committee of the University of Patras (Ref. No. 11584/6 March 2018). After obtaining the written informed consent, two healthy non-smoking male individuals (less than 30 years), who were not exposed to radiation, were not under any drug treatment and did not have any viral infection in the recent past, were used as blood donors to establish whole blood lymphocyte cultures.

#### 2.5.2. CBMN Assay Application

CBMN assay was performed according to OECD 487 Guideline [17]. Briefly, 0.5 mL of whole blood, 6.5 mL Ham’s F-10 medium, 1.5 mL FBS and 0.3 mL PHA for the stimulation of cell division were added to the culture. Then, 24 h after the beginning of incubation, cell cultures were treated with 10, 50 and 100 μg mL^−1^ of each extract S1-S4 with or without the presence of MMC (0.5 μg mL^−1^). Cytochalasin-B (at a final concentration of 6 μg mL^−1^) was added to each culture 44 h after the initiation of the experiment [25]. Cell cultures were incubated (Thermo Scientific Incubator, Thermo Fisher Scientific, Waltham, MA, USA) under 5% CO_2_ at 37 °C for 72 h in total. Subsequently, cells were harvested and collected via centrifugation (2500 rpm, 10 min), treated at room temperature with a mild hypotonic solution (Ham’s medium and Milli-Q water, ratio 3:1) and fixed with freshly prepared methanol/acetic acid (ratio 5:1, in triplicate). Finally, cells were stained with 10% *v*/*v* Giemsa and prepared for analysis. The whole experimental procedure was performed in triplicate in each case.

**Figure 1:**
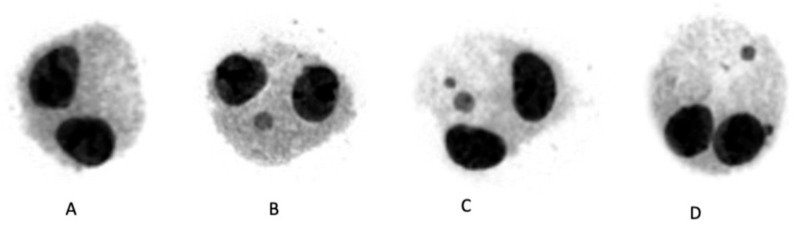
Representative photos of binucleated cells stained with Giemsa (Metafer system); (**A**) binucleated cell, (**B**) binucleated cell with one MN, (**C**,**D**) binucleated cells with two MN.

*CBPI* was evaluated by counting at least 1000 cells for each experimental point via the following equation:

$$CBPI=\frac{\left[N_{1}+2N_{2}+3(N_{3}+N_{4})\right]}{N}$$

where *N*_1_, *N*_2_, *N*_3_ and *N*_4_ correspond to the numbers of cells with one, two, three, and four nuclei and *N* is the total number of cells [26]. Evaluation of the MN frequency in at least 2000 binucleated (BN) cells (Figure 1) per concentration was conducted automatically following well established criteria [27,28], using the MNScore slide-scanning platform of the Metafer system (MetaSystems, Altlussheim, Germany).

### 2.6. Statistical Analysis

Data are expressed as mean ± standard deviation (SD) from 3 independent experiments in all cases. Statistical analyses were performed using the SPSS 25 (IBM Inc., Armonk, NY, USA, 2019) software package. Data sets were checked for the assumptions of normality (Shapiro–Wilk W Test) and of variance homogeneity (Levene’s test). Due to violation in both cases, the significant differences among variables obtained in control and challenged cells were assessed non-parametrically, using the Mann–Whitney u-test (*p* < 0.05).

### 2.7. E. arvense L. Extract Characterization by ICP-MS/MS, GC-MS and UHPLC-MS

The most beneficial extract (S4, EtOH-W) was fully characterized using mass spectrometry techniques i.e., ICP-MS, GC-MS and UHPLC-MS.

#### 2.7.1. ICP-MS/MS Analysis

The minerals of the S4 (EtOH-W) extract were determined by an Agilent 8900 Triple Quadrupole ICP-MS/MS (Agilent Technologies, Tokyo, Japan) with an Agilent SPS4 Autosampler and an Agilent Integrated Sample Introduction System (ISIS), based on ISO 17294-2 testing protocols [29,30]. For the quantification, calibration curves (R^2^ < 0.999) were created after appropriate dilution of multi-element and single-element stock solutions. The calibration standards and samples were finally prepared in an acid matrix of 2.5 % *v*/*v* HNO_3_ and 0.5% *v*/*v* HCl. The concentration (C) of the minerals were finally expressed as dry weight in μg g^−1^ according to the following formula [31]:

C = [(a × V × F)/m]

where a is the content of the mineral in the tested solution (μg L^−1^), V is the volume of the digestion solution (L), F is the dilution factor of the tested solution (if needed), and m is the initial sample mass (g).

#### 2.7.2. GC-MS Analysis

GC-MS analysis was performed on an Agilent 5975B inert MSD coupled to an Agilent 6890N Network GC system (Agilent Technologies, Santa Clara, CA, USA). An Agilent HP-5MS of 30 m length, 250 μm diameter and 0.25 μm film thickness analytical column was used. The injection volume was 2 μL. The sample was injected in splitless mode, and the injector temperature was 300 °C. The column temperature program was: 50 °C, 2 min, followed by ramp of 10 °C/min, up to a final temperature of 280 °C, 40 min. The total run was 65 min. The MSD detector acquisition parameters were as follows: the transfer line temperature 280 °C, the MS source temperature 230 °C, and the MS quadrupole temperature 150 °C. The electron impact mass spectra were obtained at 70 eV of ionization energy. NIST MS Search 2.0 software (chemdata.nist.gov, accessed on 27 May 2022, Mass Spectrometry Data Center, National Institute of Standards and Technology, Gaithersburg, MD, USA) was used for compound identification.

#### 2.7.3. UHPLC-MS Analysis

UHPLC-MS analysis was performed on an Ultimate 3000 RSLC System (Thermo Fisher Scientific, Waltham, MA, USA) coupled to an amaZon SL ion trap mass spectrometer from Bruker with an ESI source. An Acclaim RSLC 120 C18, 2.2 μm 120 Å (2.1 × 150 mm) column thermostated at 30 °C was used for the separation. Water with 0.1 % aqueous formic acid and acetonitrile was used as mobile phase A and B, respectively, at 0.3 mL/min. The following gradient program was used: 5% B (0 min), 5% B (2 min), 11% B (20 min) 24% B (33 min), 90% B (40 min), followed by a column cleaning and re-equilibration step. The injection volume was 20 μL. Full scan MS spectra were acquired in both positive and negative mode. For the determination of the chemical composition of the extracts, the negative ion ESI mass spectra were used. The identification was based on the interpretation of their MS spectra (molecular and fragment ions) as well as on the literature data [32].

## 3. Results

### 3.1. Extraction Yields (Soxhlet Extraction)

The extraction yield is defined as the amount of solute extractable by the solvent at the established extraction conditions and indicates the process efficiency. The overall Soxhlet yields were obtained after 6 h, using different solvents (Table 1). As expected, the yield was higher following the increase in the solvent polarity. The highest extraction yield was observed in water-extracts (S3, 24.9 ± 0.8 wt%), while those obtained in EtOH-W and MeOH extracts (S4 and S1, respectively) were 15.2 ± 0.4 and 15 ± 0.4 wt%. The lowest yield was observed in EtOH (S2) extract, reaching a value of 9.8 ± 0.5 wt%.

### 3.2. Total Phenolic Content, Total Flavonoid Content, and Antioxidant Activity

#### 3.2.1. Total Phenolic and Flavonoid Content

According to the results, the highest TPC value is attributed to the EtOH (S2) extract (257 ± 29 mg GAE g^−1^), followed by the EtOH-W (S4) (220 ± 12 mg GAE g^−1^) and MeOH (S1) extracts (196 ± 3 mg GAE g^−1^), respectively (Table 2), while the water-extract (S3) exhibited the lowest TPC values (63 ± 1 mg GAE g^−1^).

In the case of TFC, the EtOH (S2) extract exhibited the highest value (168 ± 14 mg CE g^−1^), followed by the EtOH-W (S4) (131 ± 9 mg CE g^−1^) and MeOH (S1) (130 ± 4 mg CE g^−1^) extracts. Similarly to the TPC results, the water-extracts (S3) showed the lowest TFC (36 ± 1 mg CE g^−1^).

#### 3.2.2. Antioxidant Activity

The antioxidant activities (AA) of all *E. arvense* L. extracts were identified via ABTS, DPPH and FRAP assays (Table 3). According to the results, the EtOH (S2) extract exhibited the highest AA in all assays. The MeOH (S1) and EtOH-W (S4) extracts demonstrated high AA, with the EtOH-W (S4) extract showing higher values in ABTS and FRAP assays, while a higher value was acquired on the DPPH assay for the MeOH (S1) extract. The water (S3) extract had the lowest AA, compared to the other three extracts in all cases.

### 3.3. CBMN Assay in Human Lymphocytes

**Figure 2:**
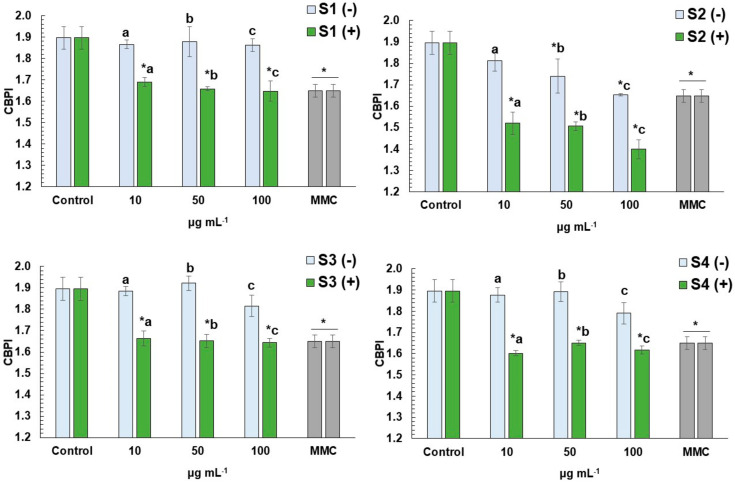
Cytotoxicity (in terms of CBPI values) of MeOH (S1), EtOH (S2), water (S3) and EtOH-W (S4) extracts of *E. arvense* L. in human lymphocytes treated with (+) or without (−) the presence of mitomycin C (MMC, 0.5 μg mL^−1^). The results are mean ± SD from 3 independent experiments in each case. Values that share the same letter differ from each other. Asterisks (*) indicates significant difference from control in each case (Mann–Whitney u-test, *p* < 0.05).

**Figure 3:**
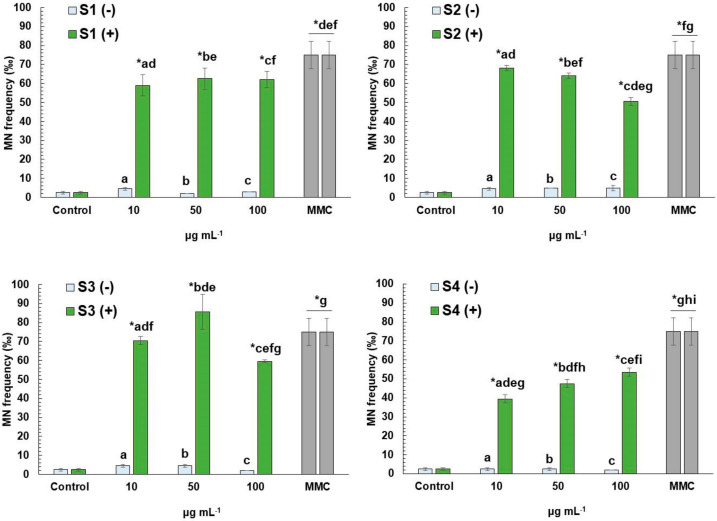
Micronuclei formation in human lymphocytes treated with MeOH (S1), EtOH (S2), water (S3) and EtOH-W (S4) extracts of *E. arvense* L. with (+) or without (−) the presence of mitomycin C (MMC, 0.5 μg mL^−1^). The results are mean ± SD from 3 independent experiments in each case. Values that share the same letter differ from each other. Asterisks (*) indicates significant difference from control in each case (Mann–Whitney u-test, *p* < 0.05).

All *E. arvense* L. extracts were tested for their cyto-genotoxic potential at three different concentrations (10, 50 and 100 μg mL^−1^), as well as their ability to attenuate MMC-mediated cytotoxic and genotoxic effects on human lymphocytes (Figure 2 and Figure 3). The cytotoxic activity of all four extracts with and without MMC was assessed via the determination of CBPI (Figure 2). Lack of cytotoxicity was observed in the case of MeOH, water-, and EtOH-W extracts (S1, S3 and S4, respectively), while EtOH (S2) extract was cytotoxic at concentrations of 50 and 100 μg mL^−1^. On the other hand, high cytotoxicity was recorded in MMC-treated cells, with or without the presence of each extract in all concentrations tested (10–100 μg mL^−1^).

Micronuclei (MN) formation observed in cells treated with different concentrations of S1–S4 extracts was almost similar (*p* > 0.05) to that observed in control cells (Figure 3), thus indicating the absence of genotoxic potential in all cases. On the other hand, MMC-treated cells with or without the presence of each extract in all concentrations tested showed high MN formation, compared to control cells. However, in cells treated with S1 and S4, at concentrations ranging from 10 to 100 μg mL^−1^, significant attenuation of MMC-mediated genotoxic effects was revealed, while similar findings were recorded for S2 (50 and 100 μg mL^−1^) and S3 (100 μg mL^−1^).

### 3.4. E. arvense L. S4 Extract Characterization by ICP-MS/MS, GC-MS and UHPLC-MS

Considering that *E. arvense* L. is rich in minerals, various microelements (Mg, Si, K, Ca, P, Mn, Cu, Zn) were determined and subsequently quantified in S4 extract, which exhibited the most beneficial effects, by ICP-MS/MS analysis. In Table 4, the concentrations of Mg, Si, K, Ca, P, Mn, Cu and Zn determined by ICP-MS/MS in S4 extract are presented. K was found to be the most abundant, reaching the value of 187,136.1 μg g^−1^. Results of microelements also revealed high levels of Ca, Mg, Si and P, whereas lower levels of Zn, Mn, and Cu were detected.

The chemical composition of the S4 extract was further studied in detail by GC-MS and UHPLC-MS analysis and 14 compounds (Table 5) were identified. Half of the compounds were identified by GC-MS and the elucidation was based on the comparison of the obtained spectra (Figure S1) with the existed in NIST library. Regarding UHPLC-MS analysis, the structural assignment of the seven compounds was performed on the basis of their molecular mass ion formation ([M-H]^−^) and their fragments (where available) compared to the data previously reported in the literature. The mass spectra of the identified compounds are depicted in Figure S2. The results suggested mainly the presence of flavonoids, flavonoids-*O*-glycosides, phytosterols, phenolic and fatty acids.

## 4. Discussion

Plant extracts are widely used for the production of novel therapeutic drugs against a number of ailments [1,2]. Solvents, such as ethanol, methanol and water, are extensively used for the extraction of valuable compounds of different origin [10,35,36,37,38]. The results of the present study show that these solvents were very effective and could mediate the beneficial effects of the plant *E. arvense* L., at least in the case of its cytogenotoxic potential.

### 4.1. Characterization of Extracts

According to the results, the solvent with the highest polarity (water) led to the highest yield of the *E. arvense* L. extract, whereas with ethanol the lowest yield was obtained. Similar values were obtained in the case of methanol and ethanol-water at approximately 15%. Those findings are in accordance with previous studies, showing that solvents with high polarity could lead to higher extraction yields [4,39]. Moreover, our study showed that all extracts exhibited satisfactory and relatively high TPC and TFC values, following the order S2 > S4 > S1 > S3 (EtOH > EtOH-W > MeOH > water extracts, respectively), with the observed values being within the range previously reported by Cetojevic-Simin et al. [6]. Similarly, Uslu et al. [40] reported high levels of TPC in *E. arvense* L. EtOH extracts, while Nagai et al. [41] demonstrated that the aqueous extract of *E. arvense* L. had lower TPC values than those observed in EtOH extracts.

Since the Soxhlet technique can recover bioactive compounds from natural products, without affecting the bioactivity of the extracted compounds [11], all extracts obtained in this study were evaluated in terms of their antioxidant activity, using the ABTS, DPPH and FRAP assays. The results showed differences among the extracts, with the highest AA values observed in EtOH (S2) extract, followed by EtOH-W and MeOH extracts (S4 and S1, respectively), while the lower values were observed in the water extract (S3), which is in accordance with Mimica-Dukic et al. [42]. Thus, the obtained TPC and TFC values of the different extracts and their AA were further reinforced by previous evidence regarding the relationship among antioxidant efficiency of extracts containing phenolics and flavonoids [40,43,44,45]. Differences between our TPC, TFC and AA values and those previously mentioned could be due to different factors such as geographical and environmental parameters, pretreatment (freeze-drying) of the raw material, storage conditions, the particle size of said raw material, the solvent used, the solvent-to-solid ratio, the temperature, and the duration of the extraction [46,47,48].

### 4.2. Cyto-Genotoxic Potential of Extracts

The cytotoxic, genotoxic and antigenotoxic potential of *E. arvense* L. extracts (MeOH, EtOH, water, EtOH-W/S1, S2, S3, S4, respectively) was assessed via the CBMN assay, which is widely used for the investigation of the toxic profile of various compounds, via the detection of micronuclei (MN) in the cytoplasm of interphase human lymphocytes [17]. MN formation, due to the inability of acentric chromosome fragments or whole chromosomes to migrate to the poles during the anaphase stage of the cell, renders the CBMN assay appropriate for the detection of both aneugenic and clastogenic effects in challenged cells that have undergone cell division. Furthermore, the CBPI can also be calculated to identify the possible cytotoxic effects of the compound tested in each case [17,49].

As far as the cytotoxic potential of *E. arvense* L. extracts is concerned, the present study showed that MeOH, water and EtOH-W extracts (S1, S3 and S4, respectively) were not cytotoxic in all concentrations tested, while EtOH extract (S2)-mediated cytotoxicity was significant in almost all cases. In accordance with the latter, Trouillas et al. [50] reported that only high concentrations of water-soluble fraction of *E. arvense* L. could induce cytotoxic effects on B16 mouse melanoma cells, while Alexandru et al. [51] reported that *E. arvense* L. water extract was able to induce apoptosis in human leukemia cell line U937 at concentrations 4-fold higher than those currently tested. On the other hand, the EtOH extract-mediated cytotoxic effects seemed to be in accordance with previous studies. Specifically, it has been reported that EtOH extracts of *E. arvense* L. demonstrated a dose-dependent decrease in the proliferation of human peripheral lymphocytes [32], and pancreatic carcinoma AsPC-1 cells [52]. Similarly, cell viability was significantly reduced in human blood cells treated with EtOH *E. arvense* L. extracts [53], while there is evidence that EtOH-W *E. arvense* L. extracts could be cytotoxic in A549 lung carcinoma cells [54]. Uslu et al. [40], applying a response surface methodology to identify the optimal conditions for *E. arvense* L. extraction, reported that EtOH-W extracts, demonstrating high levels of AA and TPC, were highly cytotoxic against the NIH3T3 mouse fibroblast cell line, which was also revealed by the results of the present study. Interestingly, the lack of MN formation in cells treated with different concentrations of all the obtained extracts indicates the absence of genotoxic potential in all cases. These results are in accordance with previous findings in different biological models, such as human lymphocytes and *Drosophila* [53,55].

### 4.3. Cytoprotective and Antigenotoxic Effects of E. arvense L. Extracts against Mitomycin C

A different subgenus of *Equisetum*, i.e., *E. myriochaetum*, did not exert genotoxicity neither in vitro via the MN assay in human lymphocytes nor in vivo when the wing spot test in *Drosophila melanogaster* was applied [55]. Milovanović et al. [56] demonstrated a genotoxic potential of the EtOH-W extract of *E. arvense* L. against human lymphocytes, which however could be due to different geographical and environmental factors such as the soil composition. Even though an increase in MN frequency was observed in human lymphocytes treated with EtOH extract of *E. arvense* L. at 100 μg mL^−1^, the induction was not deemed statistically significant [53].

Kour et al. [57] previously examined the protective effect of the EtOH extract of *E. arvense* L. against cyclophosphamide, a chemotherapeutic alkylating agent, in the bone marrow cells of Swiss albino mice using the chromosome aberration (CA) assay in vivo. A clear antimutagenic and anticlastogenic action of the extract was observed against the mutagenic activity induced by cyclophosphamide. Other subgenuses of *Equisetum*, namely *E. herba* and *E. myriochaetum*, exerted antimutagenic and radioprotective activity when tested in somatic cells of *Drosophila melanogaster* and irradiated human lymphocytes, respectively [58,59]. Moreover, the higher TPC and TFC values found in the EtOH and EtOH-W (S2 and S4) extracts in most cases compared to the other extracts could partly explain the antigenotoxic activity exerted by them. In fact, the beneficial and medicinal properties of plants are associated with the presence of secondary metabolites. EtOH-W (S4) was the extract that showed the highest rates of antigenotoxicity at all the tested concentrations.

The observed trend can be correlated with the chemical composition of the extract and mainly the identified phenolic compounds, flavonoids and flavonoids-*O*-glycosides. The main constituents were found to be n-hexadecanoic acid (palmitic acid), oleic acid, hexadecanoic acid,14-methyl-, methyl ester, stigmasta-5,24(28)-dien-3ol (isofucosterol), γ-Sitosterol (fucosterol), (f) stigmastan-3,5-diene, campesterol, caffeic acid, quercetin-3,7-di-*O*-glucoside, kaempferol-3,7-di-*O*-glucoside, kaempferol-3-*O*-rutinoside-7-*O*-glucoside, kaempferol-3-*O*-sophoroside, kaempferol-3-*O*-glucoside and kaempferol. Phenols and flavonoids have been associated with antioxidant, anti-inflammatory, anticancer and hepatoprotective activities [60]. According to the results of Varga et al. [61], kaempferol and caffeic acid, which were also identified in this study, as well as quercetin and chlorogenic acid, were the predominant polyphenols and flavonoids found at the EtOH extract of *E. arvense* L. All the aforementioned compounds have been found to possess antigenotoxic properties in previous studies [62,63,64,65].

Anti-proliferative effects against various cancers cells including melanoma, colorectal, ovarian and breast cancer have been reported for kaempferol and its glucoside, kaempferol-3-*O*-glucoside [66].

The fatty acid components of the EtOH-W (S4), oleic and palmitic acid, are known for their antimutagenic/anticlastogenic properties. Palmitic acid showed antimutagenic activity in rats [67]. Nadathur et al. [68] have reported the antimutagenic properties of palmitic acid against the mutagen methylnitronitrosoguanidine and its mechanism of action, which is the entrapment of the mutagen into the micelles formed by the fatty acid. Both fatty acids, oleic and palmitic acid, have also demonstrated antioxidant effects [69,70].

Furthermore, the potential beneficial effects of the determined minerals i.e., Mg, Si, K, Ca, P, Mn, Cu, Zn, should also be considered. It is documented that the interactions and synergistic effects between the various components that co-exist in natural products are correlated with their potential positive impact [13,71,72].

It is worth noting that this extract had the highest ability to reduce the effect of mitomycin C at a percentage of 45% at the lowest concentration. Similar results have been published about products from the endemic Greek plant *Pistacia lentiscus var. Chia*; as has been proven in previous studies, the lowest concentrations exhibited the desirable effects whereas the highest doses could at times exert a negative effect [13,73]. This phenomenon, called hormesis, is described as the ability of a specific compound to induce opposite effects at different doses and has been widely observed in phytochemicals [74]. Furthermore, quercetin and kaempferol, two of the main compounds of *E. arvense* L. EtOH extract, have been identified as possessing hormetic effects, inducing biphasic dose-responses on cells according to their concentrations [75]. According to our results, kaempferol was found as a main constituent in EtOH-W (S4) extract.

## 5. Conclusions

The growing interest for health benefits attributed to a wide range of plants and their extracts, in addition to their economic value, has increased the need for their extensive research. In the present study, the antioxidant and cytotoxic profile as well as the genotoxic and antigenotoxic potential of four extracts of *E. arvense* L. were investigated. According to the results, the EtOH (S2), EtOH-W (S4) and MeOH (S1) extracts possessed the highest content of phenols and flavonoids, while also exhibiting the most pronounced antioxidant activities. Amongst them, the EtOH (S2) extract induced the highest cytotoxic effects in accordance with its TPC, TFC and AA values. The most important finding of remarkable novelty of the present research is the significant antigenotoxic potential demonstrated by the EtOH-W (S4) extract in all concentrations, with the lowest dose exhibiting the strongest effect, while the EtOH (S2) extract led to a dose-dependent decrease of MN frequency which was significant at the highest concentration. EtOH-W (S4) extract was found to be rich in flavonoids, flavonoid-*O*-glycosides, phytosterols, phenolic and fatty acids, as well as in minerals and mainly in K, Ca, Mg, Si and P.

Our results suggest the importance of the extraction solvent used in the resulting beneficial properties of *E. arvense* L. Furthermore, the antioxidant, cytotoxic and antigenotoxic effects exhibited by the specific extracts could lead to their use in a variety of medicinal applications and render them as valuable candidates in the health field. Plant-derived extracts, such as the studied extracts, may moderate genotoxicity as well as the adverse side effects of anti-cancer drugs, such as MMC, and contribute to the alleviation of negative effects induced by various chemotherapeutic compounds.

## Figures and Tables

**Table 1:** Soxhlet extraction yields of *E. arvense* L. using different solvents. The yield values (%) are expressed as mean ± SD from 3 independent measurements in each case. Values that share the same letter differ from each other (Mann–Whitney u-test, *p* < 0.05).

Extraction	Polarity ^#^	Time (min)	Yield (%) *
S1	6.6	360	15.0 ± 0.4 ^ac^
S2	5.2	360	9.8 ± 0.5 ^bce^
S3	10.2	360	24.9 ± 0.8 ^abd^
S4	7.1	360	15.2 ± 0.4 ^de^

S1: MeOH; S2: EtOH; S3: water; S4: EtOH-W extracts (ratio 4:1) of *E. arvense* L. * Extraction yield expressed in wt% (mean ± standard deviation) based on experiments conducted in triplicate. ^#^ References [33,34].

**Table 2:** Total phenolic content (TPC) and total flavonoid content (TFC) of *E. arvense* L. extracts with four different solvents. The results are expressed as mean ± SD from 3 independent measurements in each case. Values in each column that share the same letter differ from each other (Mann–Whitney u-test, *p* < 0.05).

Extract	TPC (mg GAE g^−1^)	TFC (mg CE g^−1^)
S1	196 ± 3 ^ab^	130 ± 4 ^ab^
S2	257 ± 29 ^ac^	168 ± 14 ^acd^
S3	63 ± 1 ^bc^	36 ± 1 ^bc^
S4	220 ± 12	131 ± 9 ^d^

S1: MeOH; S2: EtOH; S3: water; S4: EtOH-W extracts (ratio 4:1) of *E. arvense* L.

**Table 3:** Antioxidant activities (AA) of *E. arvense* L. extracts by ABTS, DPPH, and FRAP assays. The results are expressed as mean ± SD from 3 independent measurements in each case. Values in each column that share the same letter differ from each other (Mann–Whitney u-test, *p* < 0.05).

Extract	AA (μmol TE g^−1^)		
	ABTS	DPPH	FRAP
S1	1292 ± 27 ^ab^	1847 ± 158 ^a^	834 ± 34 ^a^
S2	1788 ± 221 ^ac^	2217 ± 272 ^b^	979 ± 111 ^b^
S3	243 ± 1 ^bcd^	374 ± 13 ^abc^	202 ± 4 ^abc^
S4	1417 ± 141 ^d^	1581 ± 401 ^c^	858 ± 86 ^c^

S1: MeOH; S2: EtOH; S3: water; S4: EtOH-W extracts (ratio 4:1) of *E. arvense* L.

**Table 4:** Concentrations of minerals in S4 (EtOH-W) extract.

Element	Concentration (μg g^−^^1^)
Mg	28,680.6
Si	7823.4
K	187,136.1
Ca	32,139.8
P	3988.8
Mn	257.7
Cu	237.1
Zn	1165.6

**Table 5:** Compounds identified by GC-MS and UHPLC-MS in S4 (EtOH-W) extract.

**GC/MS**	**Proposed Compound**	**Molecular Formula**	**MW**
	n-Hexadecanoic acid (Palmitic acid)	C_16_H_32_O_2_	256.4
	Oleic Acid	C_18_H_34_O_2_	282.5
	Hexadecanoic acid,14-methyl-, methyl ester	C_1__8_H_3__6_O_2_	284.5
	Stigmasta-5,24(28)-dien-3ol (Isofucosterol)	C_29_H_48_O	412.7
	γ-Sitosterol (Fucosterol)	C_29_H_5__0_O	414.7
	Stigmastan-3,5-diene	C_29_H_48_	396.7
	Campesterol	C_28_H_48_O	400.7
**UHPLC/MS**	**Proposed Compound**	**Molecular Formula**	**MW**
	Caffeic acid	C_9_H_8_O_4_	180.2
	Quercetin-3,7-di-*O*-glucoside	C_27_H_30_O_17_	626.5
	Kaempferol-3,7-di-*O*-glucoside	C_27_H_30_O_16_	610.5
	Kaempferol-3-*O*-rutinoside-7-*O*-glucoside	C_33_H_40_O_20_	756.7
	Kaempferol-3-*O*-sophoroside	C_27_H_30_O_16_	610.5
	Kaempferol-3-*O*-glucoside	C_21_H_20_O_11_	448.4
	Kaempferol	C_15_H_10_O_6_	286.2

## Data Availability

Data are contained within the article.

## Supplementary Materials

The following supporting information can be downloaded at: https://www.mdpi.com/article/10.3390/antiox11071393/s1, Figure S1. Mass spectra obtained by GC-MS analysis of (a) n-Hexadecanoic acid (Palmitic acid), (b) Oleic acid, (c) Hexadecanoic acid,14-methyl-, methyl ester, (d) Stigmasta-5,24(28)-dien-3ol (Isofucosterol), (e) γ-Sitosterol (Fucosterol), (f) Stigmastan-3,5-diene and (g) Campesterol; Figure S2. Mass spectra obtained by LC-MS in negative ionization mode of (a) Caffeic acid, (b) Quercetin-3,7-di-*O*-glucoside, (c) Kaempferol-3,7-di-*O*-glucoside, (d) Kaempferol-3-*O*-rutinoside-7-*O*-glucoside, (e) Kaempferol-3-*O*-sophoroside, (f) Kaempferol-3-*O*-glucoside and (g) Kaempferol.
