# Supplementary material for: Investigation of the Genotoxic, Antigenotoxic and Antioxidant Profile of Different Extracts from Equisetum arvense L
Source: Antioxidants (Basel). 2022 Jul 18;11(7):1393. doi: 10.3390/antiox11071393 (PMC9312020; doi:10.3390/antiox11071393)
Supplement: Supplementary file 1 [file antioxidants-11-01393-s001.zip › antioxidants-1802166-supplementary.pdf]

Supplementary Materials

Investigation of the Genotoxic, Antigenotoxic and Antioxidant Profile of Different Extracts from *Equisetum arvense* L.

Margarita Dormousoglou [1], Ioanna Efthimiou [1,2], Maria Antonopoulou [1], Damian L. Fetzer [3], Fabiane Hamerski [3], Marcos L. Corazza [3], Maria Papadaki [1], Samir Santzouk [4], Stefanos Dailianis [5] and Dimitris Vlastos [5,*]

1 Department of Environmental Engineering, University of Patras, Seferi 2, GR-30100 Agrinio, Greece; m.dormousoglou@upatras.gr (M.D.); iefthimiou@upatras.gr (I.E.); mantonop@upatras.gr (M.A.); marpapadaki@upatras.gr (M.P.)

2 Hellenic Centre for Marine Research (HCMR), Institute of Marine Biology, Biotechnology and Aquaculture, Anavyssos, GR-19013 Athens, Greece

3 Department of Chemical Engineering, Federal University of Paraná, Curitiba 81531-990, Brazil; fetzer@ufpr.br (D.L.F.); fabianehamerski@ufpr.br (F.H.); corazza@ufpr.br (M.L.C.)

4 Santzouk Samir and Co. General Partnership, PANAX, Chrissostomou Smirnis 14, GR-30100 Agios Konstantinos, Greece; smsamir@otenet.gr

5 Department of Biology, University of Patras, GR-26500 Patras, Greece; sdailianis@upatras.gr

* Correspondence: dvlastos@upatras.gr; Tel: +30-2610969239

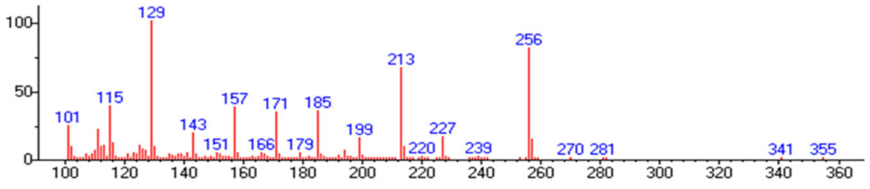

(a)

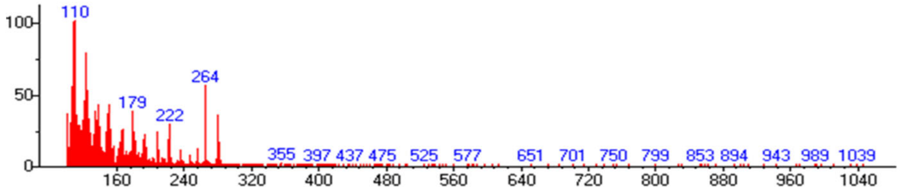

(b)

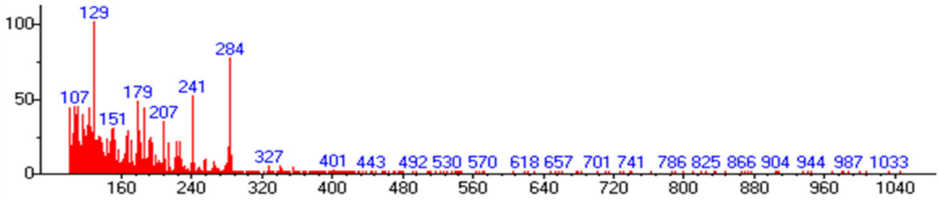

(c)

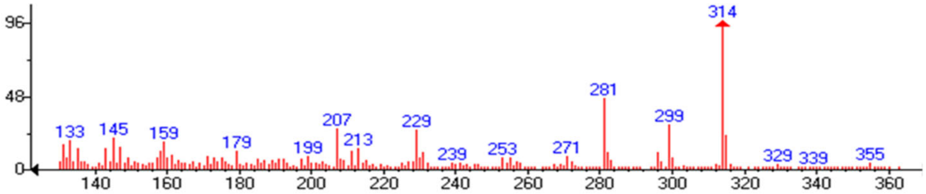

(d)

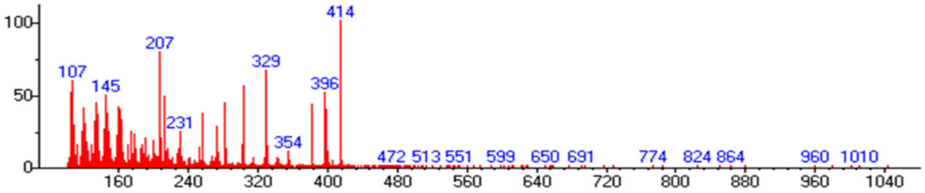

(e)

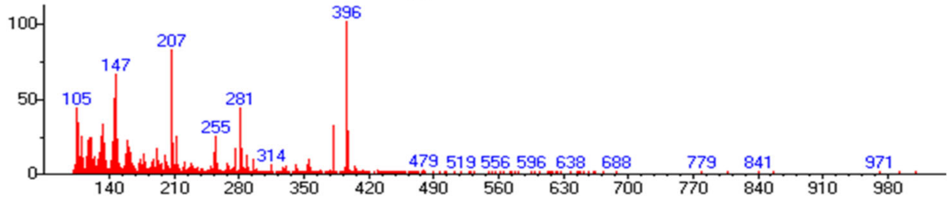

(f)

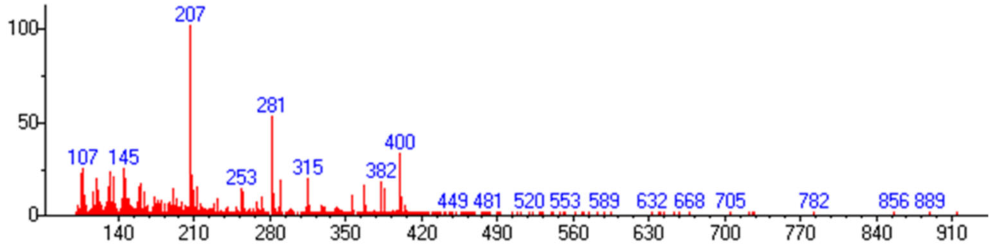

(g)

Figure S1. Mass spectra obtained by GC-MS analysis of (a) n-Hexadecanoic acid (Palmitic acid), (b) Oleic acid, (c) Hexadecanoic acid,14-methyl-, methyl ester, (d) Stigmasta-5,24(28)-dien-3ol (Isofucosterol), (e) γ-Sitosterol (Fucosterol), (f) Stigmastan-3,5-diene and (g) Campesterol.

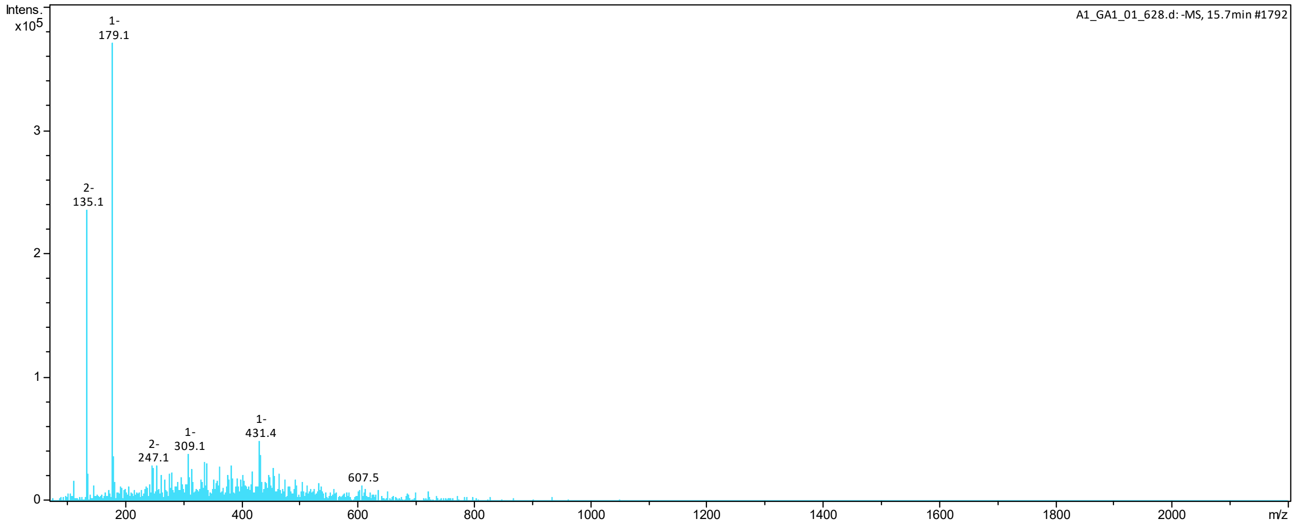

(a)

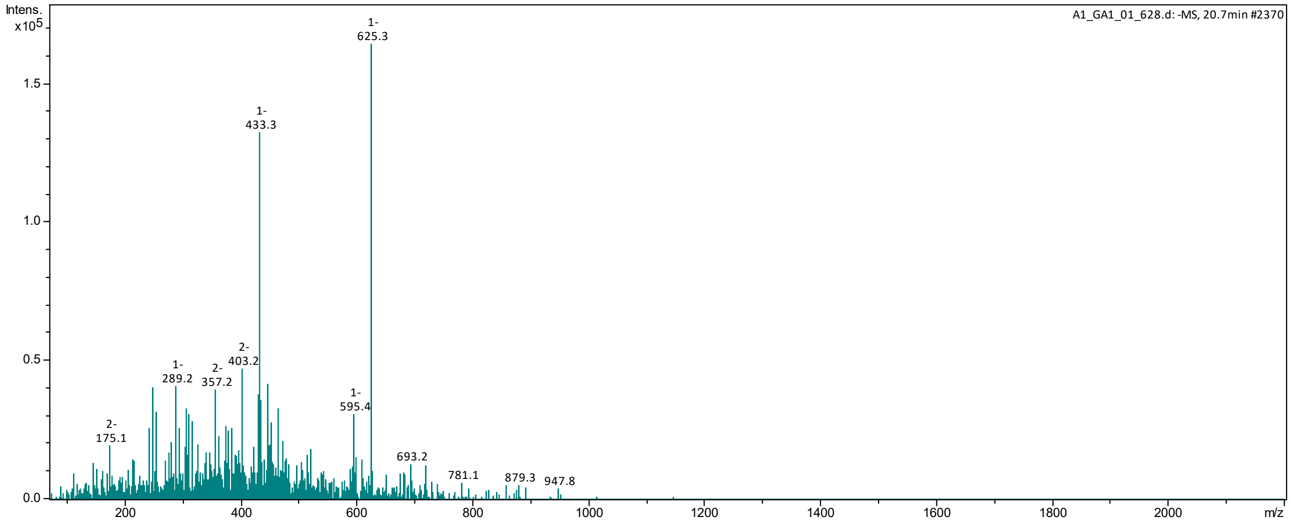
Intens.
x10^5
A1_GA1_01_628.d: -MS, 20.7min #2370
1- 625.3
1- 433.3
2- 175.1
1- 289.2
2- 357.2
2- 403.2
1- 595.4
693.2
781.1
879.3
947.8
m/z

(b)

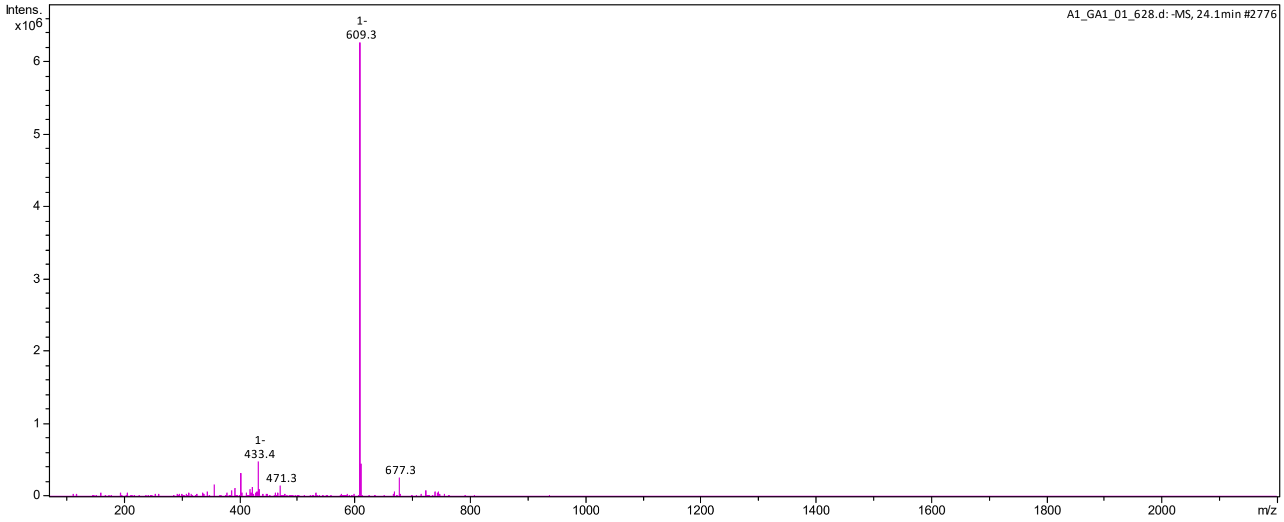
Intens.
x10^6
A1_GA1_01_628.d: -MS, 24.1min #2776
1- 609.3
1- 433.4
471.3
677.3
m/z

(c)

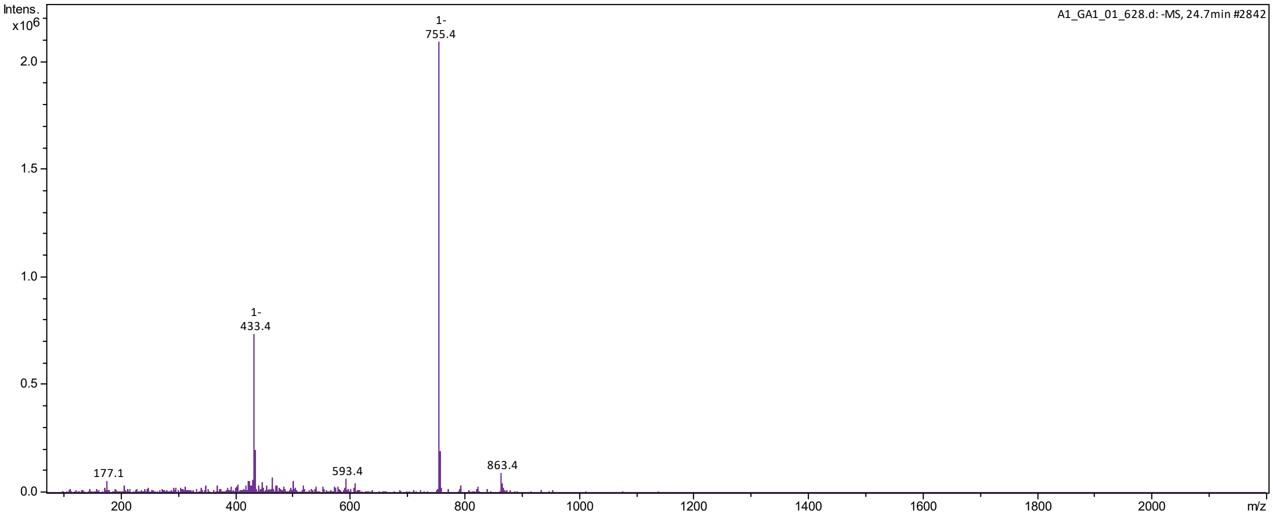
Intens.
x10^6
A1_GA1_01_628.d: -MS, 24.7min #2842
1- 755.4
1- 433.4
177.1
593.4
863.4
m/z

(d)

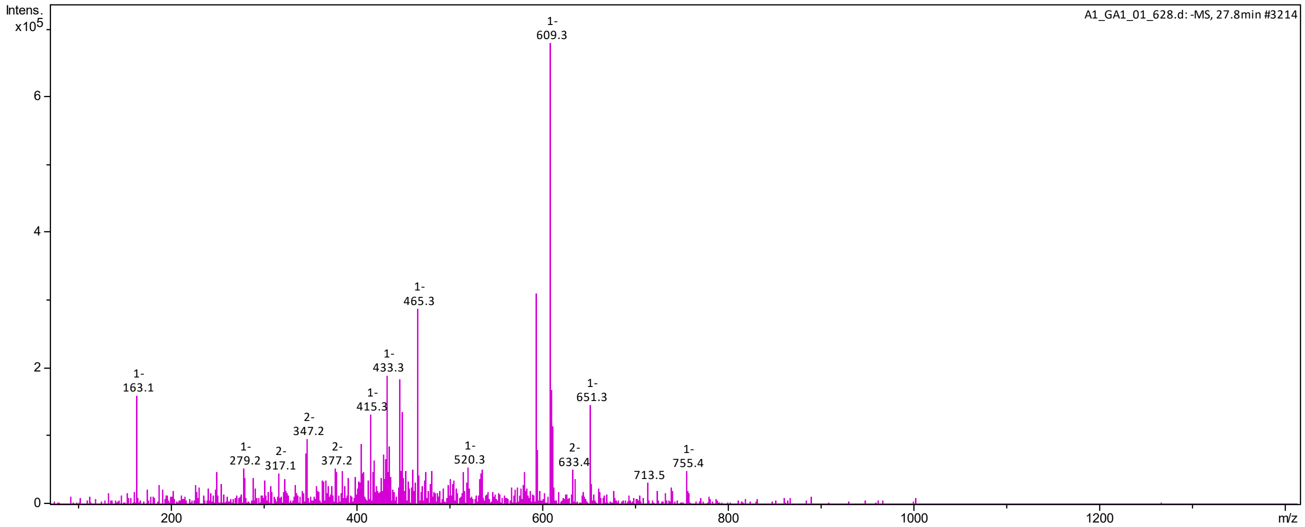
Intens.
x10^5
A1_GA1_01_628.d: -MS, 27.8min #3214
1-
609.3
1-
465.3
1-
433.3
1-
415.3
1-
163.1
2-
347.2
1-
279.2
2-
317.1
2-
377.2
1-
520.3
1-
651.3
2-
633.4
713.5
1-
755.4
m/z

(e)

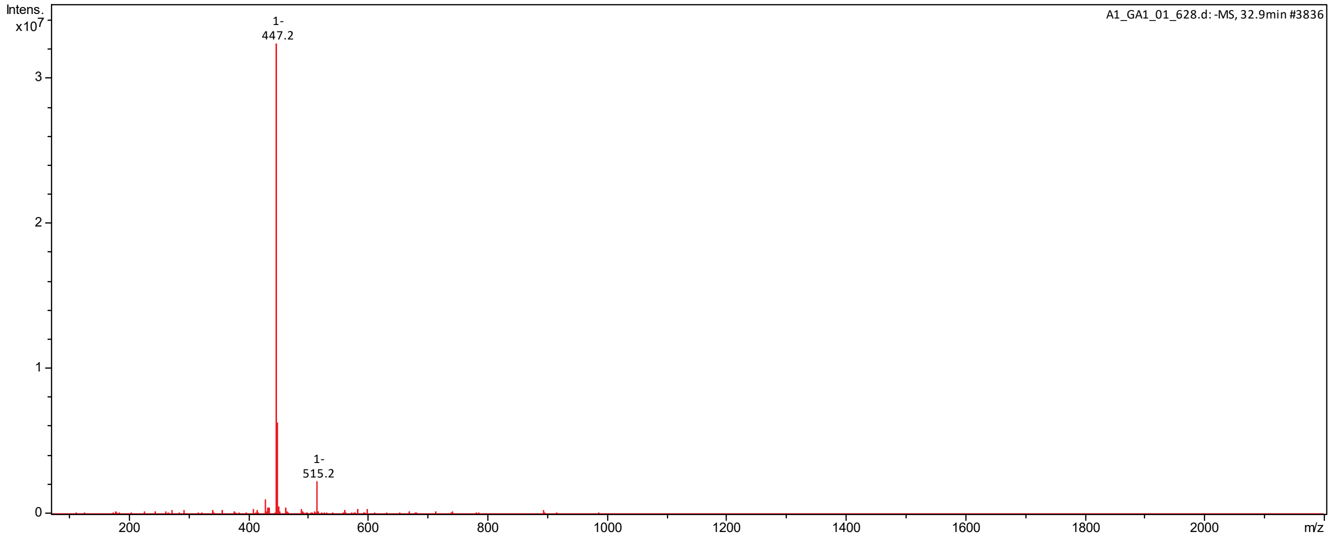
Intens.
x10^7
A1_GA1_01_628.d: -MS, 32.9min #3836
1-
447.2
1-
515.2
m/z

(f)

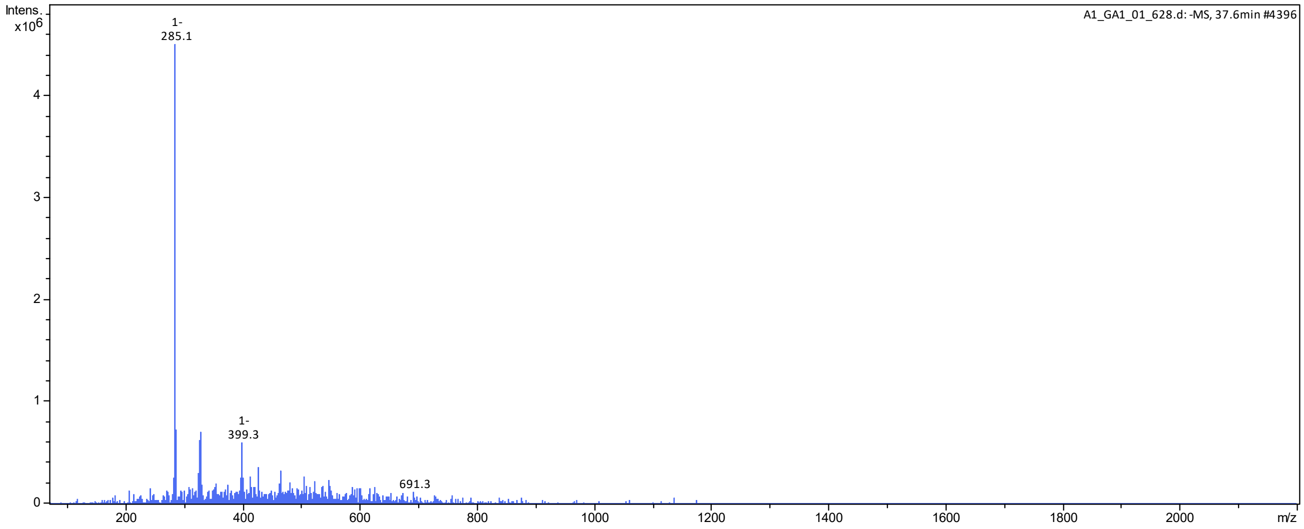

(g)

Figure S2. Mass spectra obtained by LC-MS in negative ionization mode of (a) Caffeic acid, (b) Quercetin-3,7-di-*O*-glucoside, (c) Kaempferol-3,7-di-*O*-glucoside, (d)Kaempferol-3-*O*-rutinoside-7-*O*-glucoside, (e) Kaempferol-3-*O*-sophoroside, (f) Kaempferol-3-*O*-glucoside and (g) Kaempferol.
